# Classification performance and reproducibility of GPT-4 omni for information extraction from veterinary electronic health records

**DOI:** 10.3389/fvets.2024.1490030

**Published:** 2025-01-16

**Authors:** Judit M. Wulcan, Kevin L. Jacques, Mary Ann Lee, Samantha L. Kovacs, Nicole Dausend, Lauren E. Prince, Jonatan Wulcan, Sina Marsilio, Stefan M. Keller

**Affiliations:** ^1^Department of Pathology, Microbiology and Immunology, School of Veterinary Medicine, University of California, Davis, Davis, CA, United States; ^2^College of Veterinary Medicine and Biomedical Sciences, James L. Voss Veterinary Teaching Hospital, Colorado State University, Fort Collins, CO, United States; ^3^Department of Medicine and Epidemiology, School of Veterinary Medicine, University of California, Davis, Davis, CA, United States; ^4^Independent Researcher, Malmö, Sweden

**Keywords:** machine learning, artificial intelligence, generative-pretrained transformers, Chat-GPT, text mining, feline chronic enteropathy, Real-World Evidence (RWE), Real-World Data (RWD)

## Abstract

Large language models (LLMs) can extract information from veterinary electronic health records (EHRs), but performance differences between models, the effect of hyperparameter settings, and the influence of text ambiguity have not been previously evaluated. This study addresses these gaps by comparing the performance of GPT-4 omni (GPT-4o) and GPT-3.5 Turbo under different conditions and by investigating the relationship between human interobserver agreement and LLM errors. The LLMs and five humans were tasked with identifying six clinical signs associated with feline chronic enteropathy in 250 EHRs from a veterinary referral hospital. When compared to the majority opinion of human respondents, GPT-4o demonstrated 96.9% sensitivity [interquartile range (IQR) 92.9–99.3%], 97.6% specificity (IQR 96.5–98.5%), 80.7% positive predictive value (IQR 70.8–84.6%), 99.5% negative predictive value (IQR 99.0–99.9%), 84.4% F1 score (IQR 77.3–90.4%), and 96.3% balanced accuracy (IQR 95.0–97.9%). The performance of GPT-4o was significantly better than that of its predecessor, GPT-3.5 Turbo, particularly with respect to sensitivity where GPT-3.5 Turbo only achieved 81.7% (IQR 78.9–84.8%). GPT-4o demonstrated greater reproducibility than human pairs, with an average Cohen's kappa of 0.98 (IQR 0.98–0.99) compared to 0.80 (IQR 0.78–0.81) with humans. Most GPT-4o errors occurred in instances where humans disagreed [35/43 errors (81.4%)], suggesting that these errors were more likely caused by ambiguity of the EHR than explicit model faults. Using GPT-4o to automate information extraction from veterinary EHRs is a viable alternative to manual extraction, but requires validation for the intended setting to ensure accuracy and reliability.

## 1 Introduction

Efficient, accurate, and scalable methods for extracting information from electronic health records (EHRs) are essential for conducting retrospective studies in veterinary medicine. Inaccurate information extraction can introduce bias and lead to inappropriate conclusions (1). Veterinary EHRs commonly lack standardized diagnostic codes, making automated information extraction challenging. This limitation has been identified as a barrier for leveraging routinely collected animal health data (Real-World Data) as a source for clinical evidence (Real-World Evidence) for veterinary medicine (2). Manual review, the current gold-standard for extracting information from free text, is time-consuming, tedious, and error-prone. In addition, there is a limit to the number of EHRs a human can assess, which hinders large-scale information extraction. Introducing an automated filtering step before the manual review can improve the efficiency and sensitivity of information extraction (3). Key-word searches, commonly used as a pre-filtering step, are a crude tool and risk excluding relevant EHRs. Rule-based programming (e.g., regular expressions) and supervised machine learning have shown improved classification performance over key-word searches (3). However, these methods are costly to develop, require large amounts of labeled data, and generalize poorly across different institutions and conditions. In addition, they require fine-tuning and retraining for new tasks, making them impractical for small observational studies (3).

A new and rapidly evolving tool for information extraction is the use of large language models (LLM), a form of unsupervised machine learning that can predict the next element in a text sequence after being trained on a large amount of unlabeled text (4). Early LLMs used a semi-supervised approach, where models trained on unlabeled text could be fine-tuned on labeled text for specific tasks (4). Modern LLMs can solve new tasks with few or no labeled training examples (5). However, LLMs can produce true-sounding falsehoods (hallucinations) or exhibit reasoning errors (6, 7). A recent study demonstrated good performance of GPT-3.5 Turbo for extracting information from veterinary EHRs (8). However, the performance of GPT-3.5 Turbo was not compared to that of other models, the nature of errors was not explored, and the influence of hyperparameter settings and text ambiguity were not assessed (8). Identifying the strengths, weaknesses and cost of different models is important for model selection. Understanding the cause of errors and the context in which they occur is crucial for optimizing model performance, and for setting of realistic expectations.

The objective of this study was to assess the classification performance and reproducibility of GPT-4 omni (GPT-4o) for identifying six clinical signs associated with feline chronic enteropathy (FCE) from EHRs. In addition, we compare the classification performance of GPT-4o to that of GPT-3.5 Turbo, compare the reproducibility of GPT-4o to human respondents under different conditions and investigate the relationship between human interobserver agreement and LLM errors.

## 2 Methods

### 2.1 Study design and sample size

Constructed as a retrospective cross-sectional study, the sample size was determined to estimate the sensitivity of a single test (9). The calculations accounted for a type I error rate of 5%, an acceptable margin of error of 7%, an expected sensitivity of 95% and an expected prevalence of 15%. Methods for prevalence estimation are detailed in the Supplementary material S1 and prevalence estimates for each clinical sign are available in Supplementary Table S1.

### 2.2 Case material

A test set consisting of 250 EHRs was sampled from all feline visits at the Veterinary Medical Teaching Hospital (VMTH) at University of California Davis, between 1985 and 2023. EHRs without text in the “Pertinent history field” or those used for study planning were excluded. EHRs for patients already represented in the test set were also excluded and replaced with resampled EHRs. The EHRs in the test set included only the admission date, presenting complaint, and pertinent history field from the original EHRs. The EHRs were manually deidentified by redacting possibly identifying information (see Supplementary material S2).

Pilot sets, used for initial prevalence estimation, and a tuning set, used to refine prompts and model parameters, were used during the study planning phase. These sets were distinct from the test set and are outlined in detail in Supplementary material S3 and Supplementary Figure S1.

### 2.3 Software, data type classification, scripts, and packages

The test set EHRs were analyzed using GPT-4o and GPT-3.5 Turbo (Open AI, San Francisco, CA, USA) accessed through Microsoft Azure's Open AI Application Programming Interface (API) (Microsoft Azure Redmond, WA, USA) provided by UC Davis AggieCloud Services. The account was commissioned based on a data type classification of “De-identified patient information (with negligible re-identification risk)”, a Protection Level Classification of P2 and an Availability Level Classification of A1 in accordance with UC Davis' Information Security Policy 3 (IS-3) (request RITM0074868). IS-3 is based on security standards ISO 27001 and 27002 and supports cybersecurity compliance requirements NIST 800-171, PCI, and HIPAA.

The analysis was conducted using a custom Python script (10) that leveraged the chat-completion endpoint with API version 2024-02-01. The script utilized Python's standard library (11), as well as the openai (12) and tiktoken (13) packages. Human respondents accessed the test set EHRs through a custom online survey (Qualtrics, Provo, UT, USA). All data analysis and statistical computations and visualizations were conducted using R programming language within the RStudio integrated development environment (14, 15). The custom R scripts were supported by a range of open-source packages for data science (16, 17), data import and data export (18–20), statistics (21, 22), and visualization (23–25). All scripts used in this study are available at GitHub (https://github.com/ucdavis/llm_vet_records).

### 2.4 Model tasks and prompt engineering

Respondents (humans and LLMs) were asked to perform two tasks: (1) Determine whether an EHR mentioned the presence of six clinical signs associated with FCE (classification task) and (2) cite pertinent sections of the EHR supporting the decision (citation task). Detailed instructions for both tasks were developed through iterative evaluation and adjustment (prompt engineering), informed by human and GPT-4o responses to the tuning set. The prompt used for LLM analysis only differed from the instructions provided to human respondents in the specific instructions to output the response in a JSON format.

#### 2.4.1 Classification of presence of clinical signs

The clinical signs associated with FCE were selected based on a recent diagnostic consensus statement (26) and included decreased appetite, vomiting, weight loss, diarrhea, constipation and polyphagia. The instructions specified that a “current”, or “recently present” clinical sign qualified as “present” and allowed answers were “true” or “false”. A precise time cut-off for what was considered “recent” was not provided as preliminary experiments indicated that such a criterion led to false negative results for intermittent signs (see Supplementary material S4).

#### 2.4.2 Citation of supporting text

To trace respondent decisions for classification error analysis, respondents were instructed to cite pertinent sections of the EHR. The instructions specified that only copy-pasted text should be provided, each text section should be enclosed in quotation marks, different portions of the text should be separated by white space, and ellipses should not be used to shorten the text.

### 2.5 EHR analysis

#### 2.5.1 EHR analysis by LLM

The Azure Open AI API allows setting a “temperature” value between 0 and 2, where a temperature of 0 produces the most likely response, while higher values prompt the model to generate more varied and creative outputs (27). The test set was analyzed at temperatures 0, 0.5, and 1, which were chosen based on initial experiments that showed high failure rates and invalid JSON formats at temperatures 1.5 and 2 (Supplementary Figure S2). Each analysis was repeated five times at each temperature setting. In addition to question responses and text citations, the time to complete and the cost were documented.

#### 2.5.2 EHR analysis by humans

The test set records were analyzed by five human respondents: two veterinary students (who had completed their second and third years of study, respectively), and three veterinarians (one recent graduate and two with 2-years post-graduate experience each). The humans were blinded to each other's responses and to the responses of the LLMs.

### 2.6 Assessment of classification performance

The majority opinion (mode) of human responses was considered the reference standard and the mode of the LLM responses was classified as either a true positive, false positive, true negative, or false negative. For each clinical sign, sensitivity (also referred to as “recall”), specificity, positive predictive value (PPV) (also referred to as “precision”), and negative predictive value (NPV) were calculated and reported along with 95% confidence intervals, using the “Wilson” method (28). The F1 score (the harmonic mean of sensitivity and PPV) and balanced accuracy (the arithmetic mean of sensitivity and specificity) were computed. To summarize classification performance across clinical signs, the median and interquartile range (IQR) were reported for each performance metric. The statistical significance of differences in responses between GPT-4o and GPT-3.5 Turbo at temperature 0, as well as between different temperature settings of GPT-4o was assessed with McNemar's chi square test with continuity correction and 1 degree of freedom.

### 2.7 Assessment of reproducibility

Reproducibility was analyzed for both human respondents and repeated runs of GPT-4o. Cohen's Kappa was calculated separately for each unique pair of respondents and averaged across human pairs and pairs of repeated GPT-4o runs at each temperature.

### 2.8 Assessment of compliance with instructions

Compliance with instructions was assessed in three main areas: (1) adherence to output format instructions, (2) providing a true or false response to classification questions, and (3) following citation instructions (citation compliance). The responses generated by the LLMs were assessed for all three areas, while human responses were only evaluated for citation compliance (see Supplementary material S5).

### 2.9 Assessment of classification errors

All instances where the mode LLM response differed from the majority opinion (mode) of human respondents were considered errors. If discrepant responses cited the same text sections, the cause of error was assumed to be a difference in interpretation (interpretation discrepancy). If the citations differed, it was assumed that some respondents missed relevant sections (citation discrepancy).

Additionally, errors were further categorized based on ambiguity in the alignment between the description of the clinical sign in the EHR and its definition. If the description could not be conclusively interpreted as meeting or not meeting the qualitative definition of a clinical sign, the error was classified as “qualitative ambiguity”. Similarly, if the timing or chronology described in the record could not be conclusively aligned with the temporal definition of a clinical sign (e.g., distinguishing between a historic vs. present sign), the error was classified as temporal ambiguity.

## 3 Results

### 3.1 Case material

The test set consisted of 250 EHRs from cat visits occurring between 1991 and 2023 and ranging in word length from 3 to 1262. Although all feline EHRs between 1985 and 2023 were initially considered, no EHRs prior to 1991 contained text in the “Pertinent History” section of the report and were thus excluded. The flow of EHR selection is depicted in Supplementary Figure S1.

### 3.2 Classification performance

**Figure 1:**
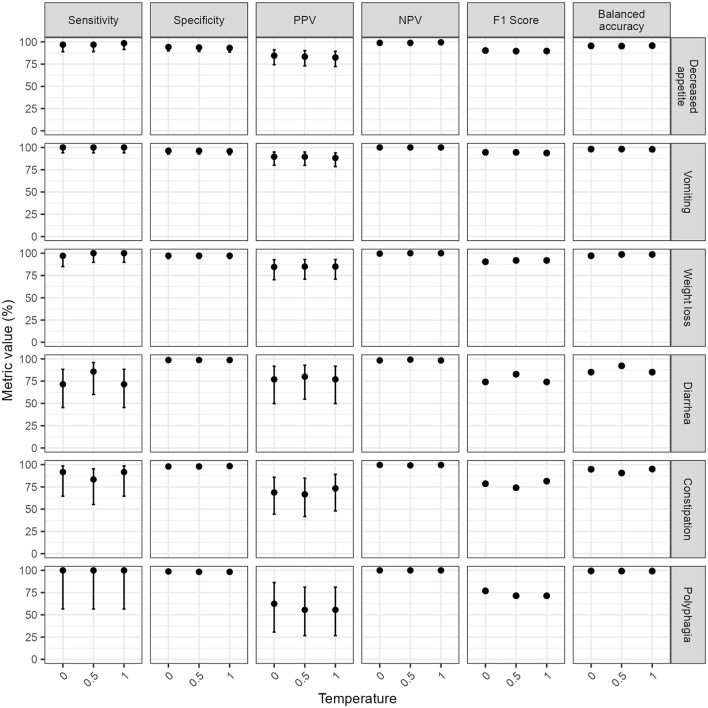
Classification performance metrics of GPT-4 omni (GPT-4o) for extracting the presence or absence of six clinical signs at different temperatures. Classification performance metrics for each clinical sign was computed by comparing the mode of GPT-4o responses from five repeated runs at each temperature to a reference standard composed of the majority opinion (mode) of five human respondents. Note the wide confidence intervals of classification performance metrics for three clinical signs of low prevalence in the test set (diarrhea, constipation, and weight loss) hindering interpretation of subtle variations of classification performance estimates across temperatures for these clinical signs. Error bars represent 95% confidence intervals. F1 scores and balanced accuracy are derivatives of sensitivity, specificity, and positive predictive value (PPV); therefore, are reported without confidence intervals. NPV, negative predictive value; PPV, positive predictive value.

The performance metrics were computed using the majority opinion of human respondents as a reference standard. The sensitivity specificity, balanced accuracy and NPV for GPT-4o averaged over 96% across clinical signs, regardless of temperature (Figure 1). The average PPV and F1 scores were lower (80.7% and 84.4% respectively at temperature 0) due to GPT-4o errors being dominated by false positives.

The temperature setting did not significantly impact the classification performance (Temperature 0 vs. temperature 0.5: χ^2^ = 0.9, *p*-value = 0.34; temperature 0 vs. temperature 1: χ^2^ = 1.07, *p*-value = 0.3, temperature 0.5 vs. temperature 1: χ^2^ = 0, *p*-value = 1). GPT-3.5 Turbo performed significantly worse than GPT-4o at temperature 0 (χ^2^ = 29.9, *p*-value < 0.0001), particularly for sensitivity (81.7%, IQR 78.9–84.8%).

The average (median) performance metrics for GPT-4o and GPT-3.5 Turbo, across all clinical signs, are reported together with interquartile ranges (IQRs) in Supplementary Table S2.

### 3.3 Reproducibility

**Figure 2:**
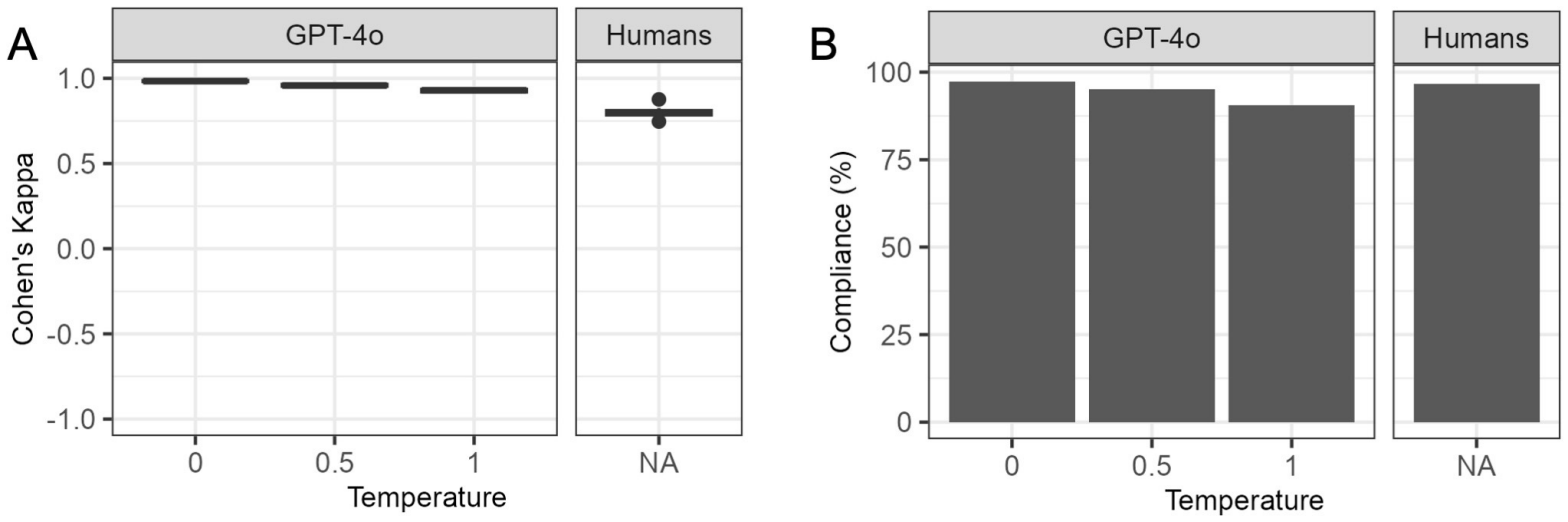
Interobserver agreement and citation compliance for humans and GPT-4 omni (GPT-4o) at different temperatures. GPT-4o, GPT-4 omni. **(A)** Interobserver agreement. Cohen's Kappa was calculated for each unique pair of human respondents, and repeated runs of GPT-4o at different temperatures. GPT-4o showed a decline in agreement between consecutive runs at higher temperatures yet maintained higher agreement than human respondents even at the highest temperature. **(B)** Citation compliance. Citation compliance was assessed by ensuring each citation was properly enclosed in quotes, separated by white-space and matched exactly with the electronic health record (EHR) text. GPT-4o had slightly higher compliance than humans at temperature 0, but its compliance decreased at temperatures 0.5 and 1, falling below that of human respondents.

The interobserver agreement of GPT-4o responses across consecutive runs decreased at higher temperature settings yet remained higher than human interobserver agreement even at the highest temperature setting (Figure 2A). The average Cohen's kappa between repeated runs of GPT-4o ranged from 0.98 at temperature 0 to 0.93 at temperature 1, while the average Cohen's kappa between human respondents was 0.8. Supplementary Table S3 contains the summary statistics as well as Cohen's Kappa per pair of respondents for human respondents and repeated runs of GPT-4o at each temperature.

### 3.4 Compliance with instructions

Unlike preliminary experiments at temperatures 1.5 and 2.0 (Supplementary Figure S1), GPT-4o produced outputs for all questions and maintained correct JSON format for over 99% of questions at temperatures 0, 0.5, and 1.0. All output with incorrect JSON format were manually corrected. GPT-4o adhered to the instructions to provide “true” or “false” answers in over 99.9% of classification questions across all temperatures. However, it responded with “NA” to one question at temperature 0 and two questions at temperature 1. Compliance with citation instructions decreased as temperatures increased for GPT-4o (Figure 2B). Human respondents complied with citation instructions less often than GPT-4o at temperature 0 but more frequently than GPT-4o at temperature 0.5 and 1. Supplementary Table S4 contains details on compliance with instructions for GPT-4o and human respondents.

Citations for both human respondents and GPT-4o at temperature 0 that did not exactly match the EHR text were manually reviewed. All discrepancies were attributed to either minor deviations in quotations, capitalization, punctation or spacing, shortening of the text, paraphrasing, or including the question or field name in the response. No hallucinations (citations not present in the EHR) were detected. Supplementary Table S5 contains details on discrepancies between citations and EHR texts.

Only one instance of a citation by GPT-4o at temperature 0 altered the meaning of the text: the EHR stated “occasionally strains in litter box to defecate, no diarrhea” but GPT-4o shortened this to “occasional diarrhea”. Despite this change, GPT-4o correctly classified the case as “false” for diarrhea, indicating that the misquotation did not affect the classification outcome.

### 3.5 Classification errors

**Figure 3:**
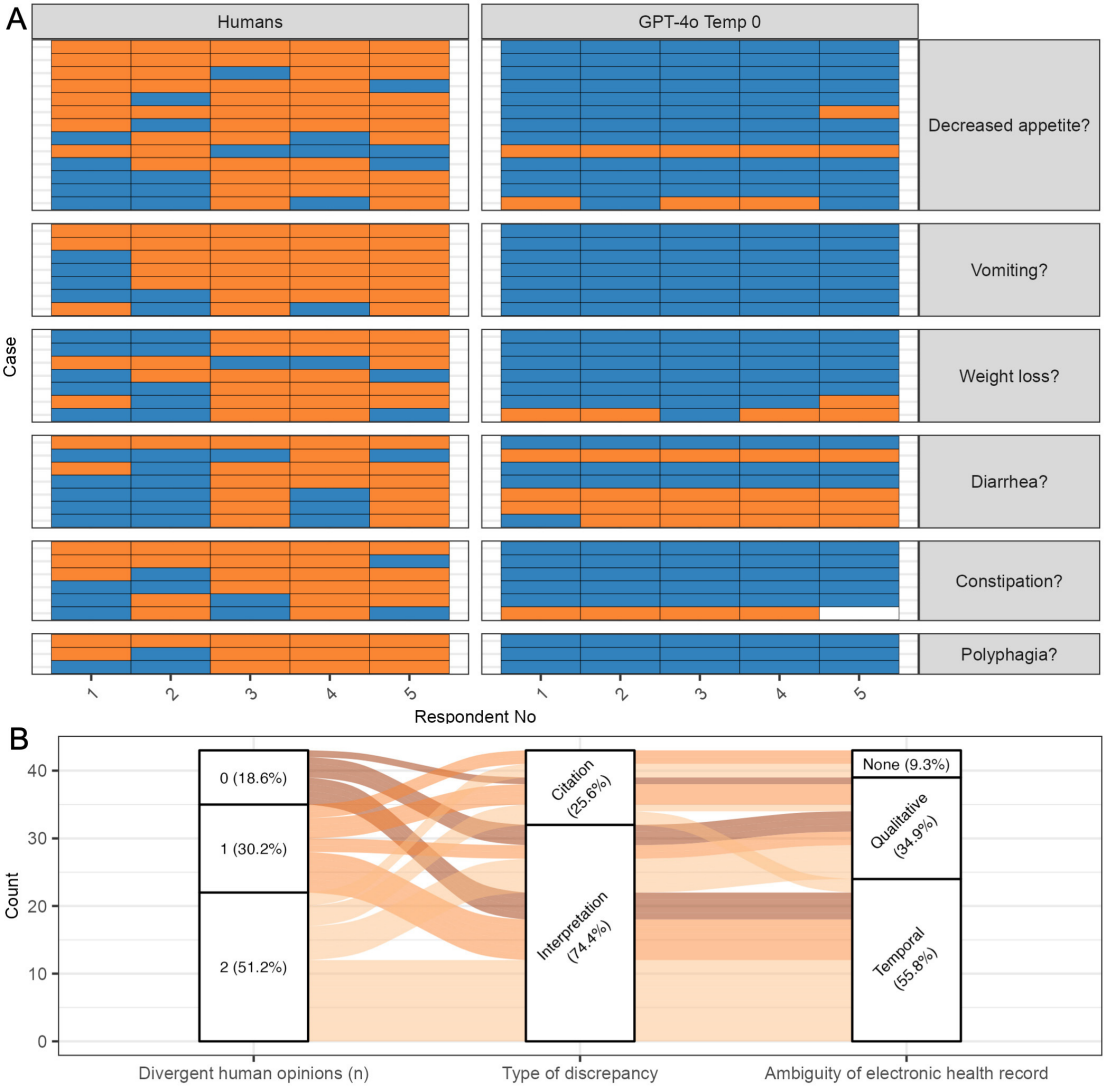
Classification errors by GPT-4 omni (GPT-4o) at temperature 0. All questions where the mode GPT-4o classification response disagreed with the majority opinion (mode) of human respondents were considered errors. **(A)** Human and GPT-4o responses. Five human respondents and five repeated runs of GPT-4o responded to questions on the presence of six clinical signs. False positive errors (instances where GPT-4o answered “true” and the majority of humans answered “false” were more common than false negative errors. Blue, true; Orange, false; white, NA; GPT-4o, GPT-4 omni; Temp, temperature. **(B)** Classification errors. Most errors occurred in questions where at least one human respondent disagreed with the majority opinion. Interpretation errors were more common than citation errors. For interpretation errors, temporal ambiguity was more common than qualitative ambiguity. Some citation errors involved electronic health records without ambiguity, suggesting that some respondents overlooked relevant sections of the text.

The mode response of repeated runs of GPT-4o at temperature 0 differed from the majority opinion response of the five human respondents in 43 out of 1,500 questions (2.9%) (Figure 3A). Most errors were false positives [35 out of 43 total errors (81.4%)]. All human and GPT-4o responses at all temperatures are depicted in Supplementary Figures S3–S8.

Most GPT-4o errors [35 out of 43 total errors (81.4%); 35 out of 1,500 questions (2.3%)] occurred in questions where at least one human assessor disagreed with the human majority, indicating potential ambiguity regarding the clinical sign in the EHR (Figure 3B). In 22 of these errors [51.2%; 22 out of 1,500 questions (1.5%)], two human respondents disagreed with the human majority opinion and sided with GPT-4o. In 13 errors [30.2%; 13 out of 1,500 questions (0.9%)], one human assessor disagreed with the majority opinion and sided with GPT-4o. Only eight errors [18.6%; eight out of 1,500 questions (0.5%)] involved GPT-4o responding incorrectly to a question where all human respondents agreed. For instance, an EHR noting “owner found a spit-up phenobarbital pill on the ground” led all GPT-4o runs to answer “true” for vomiting, while all human respondents answered “false”, not citing the text section.

Interpretation errors, i.e., errors where humans and GPT-4o cited the same text but answered differently were more common than citation errors where humans and GPT-4o cited different text (Figure 3B) [32/43 (74.4%) vs. 11/43 errors (25.6%) of total errors].

Interpretation errors arose more frequently from EHR texts with temporal ambiguity than from EHR texts with qualitative ambiguity [22/32 (68.8%) vs. 10/32 (31.2%) of interpretation errors]. For example, an EHR noting a cat was “polyphagic” at a previous visit, but “eating less” after treatment, leading to mixed responses among human respondents due to temporal ambiguity. In contrast, an EHR describing a cat with “1 bowel movement consisting of a hard, crusty piece of feces covered with a softer, outer layer” led to mixed responses among humans for constipation due to qualitative ambiguity.

While some citation errors involved EHRs with qualitative or temporal ambiguity, others did not, suggesting that some respondents overlooked relevant sections of the text (Figure 3B). For example, an EHR noting, “weight 6/30/01 16.5 lb, weight 11/00 20.5 lb” led two out of five humans (and all GPT-4o runs) to answer “true” for weight loss, while three out of five humans answered “false” without citing the text. Notably, most of these instances involved the majority of human respondents answering “false” while GPT-4o answered “true”, suggesting that some errors might reflect flaws in the human majority opinion rather than true GPT-4o errors. Only one instance involved GPT-4o missing an explicit mention of a clinical sign that the majority of humans did not, indicating that GPT-4o was better at identifying relevant portions of the text then human respondents.

### 3.6 Time and cost

GPT-3.5 Turbo analysis was quicker and cheaper than GPT-4o analysis. The median time and cost per EHR were 1.6 s (IQR 1.4–1.9 s) and 0.07 US cents (IQR 0.06–0.08 cents) for GPT-3.5 Turbo and 2.5 s (IQR 1.9–3.3 s) and 0.7 US cents (IQR 0.7–0.9) for GPT-4o.

## 4 Discussion

This study demonstrated a high classification performance of the LLM GPT-4o in identifying clinical signs consistent with FCE in EHRs from a single veterinary referral hospital. The findings indicate near perfect sensitivity and negative predictive value, an outcome favorable for the intended use of the model as a screening tool. These results align with two previous studies that analyzed the classification performance of GPT models using human manual review as the reference standard. One of the studies used GPT-3.5 Turbo to identify cases of obesity in veterinary medical EHRs, reporting a sensitivity of 100% (8), while another study with a previous version of GPT-4 (1106) achieved a 97% sensitivity in identifying comorbidities in human cancer patient EHRs (29). In both these studies, as well as the current one, errors were dominated by false positives, where the LLM indicated a clinical sign as present while the human reviewer considered it absent, resulting in lower PPVs and F1 scores.

In addition to an excellent classification performance, this study demonstrated good reproducibility, which was higher between repeated runs of GPT-4o than human respondents, regardless of temperature settings. At temperature 0, average Cohen's Kappa for repeated runs of GPT-4o approached perfect agreement, suggesting that a single analysis run is sufficient and that averaging the results over multiple runs may not be necessary at this temperature. In contrast to previous studies, this study employed multiple human reviewers, which enabled the evaluation of interobserver agreement, and the identification of challenging records characterized by low levels of consensus among reviewers.

Increasing the temperature did not negatively affect classification performance but reduced reproducibility and compliance, suggesting that lower temperature settings are better suited for this analytic task. Since the temperature setting cannot be adjusted in the web-based versions of ChatGPT, reproducing the experiments conducted in this study would require the use of an API interface.

Most GPT-4o errors occurred in instances where human respondents disagreed, suggesting that many of these errors were “reasonable interpretations” of ambiguous information. The majority of disagreements among human respondents involved temporal ambiguities. Notably, respondents were asked to assess the “presence” of a clinical sign and that a “current”, or “recently present” clinical sign qualified as “present”. Supplying a more stringent definition of “presence”, such as including time cut-offs for “current” or “recent”, might have reduced the frequency of errors. However, this approach was ultimately dismissed to avoid the systematic exclusion of chronic intermittent clinical signs, which are vital for identifying FCE.

The varying experience level of human respondents (students and veterinarians) may have contributed to interobserver variability. However, at our institution, manual review of EHRs for retrospective studies is often assigned to students, making their inclusion representative of real-world practice. In our experience students' caution and attention to detail can offset their lack of experience and may even surpass veterinary specialists in record review.

Although the classification performance of human reviewers cannot be assessed when using human reviewers as a reference standard, some discrepant responses arose from humans missing relevant sections of the text. Similarly to a previous study (29), failing to cite explicit mentions of a clinical sign was much rarer for GPT-4o than humans suggesting that in some instances, GPT-4o may exceed human sensitivity.

These findings also highlight a broader issue: human reviewers, while serving as the reference standard, are not infallible. Any discrepancies between GPT-4o and human responses may reflect differences from an imperfect standard rather than definitive model failures. To address this issue, we complemented performance metrics with a detailed error analysis. This approach provided a more nuanced understanding of presumed model errors and revealed instances where ambiguities in the EHR, rather than true model errors accounted for the observed differences.

Hallucinations, a major concern for utilizing LLMs for veterinary research (30), were not observed in this study. However, instances of paraphrasing, shortening, and non-adherence to instructions for citations occurred even at the lowest temperature setting of 0. Although these deviations did not affect the GPT-4o response in any of the observed cases, they underscore that LLMs, unlike rule-based computer programs, are probabilistic, and may not always comply with the instructions provided.

In our specific setting, GPT-4o outperformed GPT-3.5 Turbo but was 10 times more expensive to run. However, it is possible that the performances of both models are more similar when applied to other tasks. For instance, Fins et al. (8) achieved near perfect sensitivity for detection of mentions of obesity with GPT-3.5 Turbo suggesting that this more cost-efficient model may be sufficient for some applications. In addition, it is conceivable that LLMs not tested in the current study perform better than GPT-4o or perform similarly at lower cost. Open-source LLMs could be particularly appealing when evaluating large numbers of EHRs, where cost is a limiting factor.

Only EHRs from a single tertiary referral clinic were included in this study. The quality of EHRs can vary across different veterinary settings, and it is unclear if our findings would generalize to less detailed records. This highlights the importance of validating LLMs for the specific task and environment in which they are intended to be used.

In contrast to ChatGPT, the online chat version of GPT, API applications through OpenAI or Microsoft Azure can be configured to comply with federal privacy regulations for human EHRs. Previous studies using LLMs for this purpose have relied on de-identified medical records (8, 29), synthetic data (31) or locally run open source LLMs (31). While the de-identification of EHRs is feasible for a dataset size such as used in the current study, de-identifying larger sets of records might pose a significant barrier and negate most benefits of using an LLM over manual review. Although privacy and security regulations are less stringent in veterinary medicine than in human medicine, obtaining approval to use these types of applications is an essential point to consider prior to study execution.

In conclusion, the use of GPT-4o to extract information from veterinary EHRs can be a reliable alternative to human manual extraction. While considerations for cost and data privacy remain, this technology unlocks new possibilities for retrospective data analysis at a scale previously unattainable. This capability is critical for transforming routinely collected Real-World Data into Real-World Evidence to inform clinical practice and research in veterinary medicine. Future work should focus on scaling up data mining in veterinary medicine, integrating data from multiple institutions, and developing guidelines for ongoing validation and comparison of LLMs in this field.

## Data Availability

The datasets used in this study are not publicly available due to legal restrictions on sharing EHR data. However, the data can be provided upon request by contacting smkeller@ucdavis.edu.

## Supplementary material

Supplementary Presentation 1 Subheaders and text citations updated to comply with format for other supplemenrary files. The order of two subsectuons were changes to comply wiith edits of the text.

Supplementary Presentation 2 All figures compiled to a PDF. The order of Fig S1 and S2 changed (to comply wth changes in the text) Lgeneds updated to comply with format of main figures.

Supplementary Presentation 3 Saved as PDF & changes in legends to coply with format. for main paper.
