# Supplementary material for: Classification performance and reproducibility of GPT-4 omni for information extraction from veterinary electronic health records
Source: Front Vet Sci. 2025 Jan 16;11:1490030. doi: 10.3389/fvets.2024.1490030 (PMC11780673; doi:10.3389/fvets.2024.1490030)
Supplement: Supplementary Presentation 1 — Subheaders and text citations updated to comply with format for other supplemenrary files. The order of two subsectuons were changes to comply wiith edits of the text. [file Presentation_1.pdf]

Supplementary Material

Supplementary Material S1: Methods for prevalence estimation

The prevalence of each clinical sign was estimated for sample size calculation using the following methods:

- Keyword searches of the electronic health record (EHR) of all feline visits at the Veterinary Medical Teaching Hospital (VMTH) at University of California Davis, between 1985 and 2023
- Manual review of a random subset of EHRs.
- Keyword searches of all EHRs containing text in the "pertinent history field".
- Manual review of the pertinent history section in a random subset of all EHRs

Prevalence estimates for sample size calculations were based on an approximation of expected prevalence across clinical signs. The selected prevalence estimate (15%) reflects a compromise to account for feasibility, as estimates for less common clinical signs would have resulted in an unmanageable sample size.

Supplementary Table S1 contains estimated prevalences by the different methods. Note that the last row in the table represents the prevalence achieved in the study and was not available for sample size calculation.

Supplementary Material S2: Methods for de-identification of EHRs

Each EHR was assigned a consecutive case number and de-identified by manually redacting the following information:

- Patient name (replaced by "<Redacted patient name>")
- Names of other pets (replaced by "<Redacted other pet name>")
- Names of humans (replaced by "<Redacted human name>")
- Geographic locations (replaced by "<Redacted geographic location>")
- Visit numbers (replaced by "<Redacted visit number>")
- Microchip number (replaced by "<Redacted microchip number>")
- Hospital patient identification number (replaced by "<Redacted id number>")
- Other information about owner (replaced by "<Redacted other>")

Supplementary Material S3: Methods for EHR sets used for study planning

The following sets of EHRs were used for study planning:

- **"Pilot free text"**: An initial pilot set consisting of 270 EHRs sampled from all feline visits at the Veterinary Medical Teaching Hospital (VMTH) at the University of California Davis, between 1985 and 2023. Records without text in any of the fields

“Presenting complaint”, “Pertinent History”, “Physical Examination”, “Problems”, “Procedures”, “Plans and Progress Notes”, “Clinical diagnosis”, “Comments”, “Discharge Summary”, or “Discharge Instructions” were excluded. The first 34 records in this set were manually assessed by one veterinarian (JMW), documenting the presence of clinical signs mentioned in any of the fields above in a spreadsheet and recording the time taken to assess each record. The strategy of reviewing the full records was abandoned due to the length of time needed to assess each record being deemed unfeasible for the study.
- **“Pilot history”:** A pilot set consisting of 100 EHRs was sampled from records containing text in the “Pertinent history field”. Text from the “Admission date”, “Presenting Complaint” and “Pertinent History” fields were manually deidentified and used to construct a pilot survey in Qualtrics (Qualtrics, Provo, UT, USA). Through the survey, one veterinarian (JMW) documented the presence of clinical signs mentioned in the “Presenting Complaint field” or “Pertinent history” field, as well as the time used to assess each record.
- **“Tuning set”**: A tuning set, consisting of 10 EHRs, were selected from the “Pilot history” set and used for development of instructions (prompt engineering) and temperature settings.

Supplementary Material S4: Methods for development of instructions (Prompt engineering)

Detailed instructions for answering the questions and providing the reference were developed through iterative evaluation and adjustment, based on human and GPT-4o responses to 10 EHRs not used in the test set (tuning set, see Supplementary Methods for sampling of sets used for study planning). The primary focus was on refining instructions for answering true/false questions, with minor adjustments to the sections on referencing text and the example report.

The initial prompt provided the following instructions for answering true/false questions:

- “If the text indicates that the clinical sign is currently present, answer TRUE. Otherwise answer FALSE.”
- “Clinical signs occurring within the previous week should be considered current even if the clinical sign is waxing and waning, and not present at the precise moment of the visit.”

The tuning set records included explicit mentions of clinical signs (9), non-explicit mentions of clinical signs (2), abbreviations for clinical signs (3), mentions of intermittent clinical signs (3), mentions of historic clinical signs (2) and mentions of resolved but recent clinical signs (3). Using the initial prompt, the LLM response was satisfactory for explicit clinical signs, non-explicit mentions and abbreviations but discrepancies arose with intermittent, historic and resolved clinical signs. Increasingly stringent instructions (prompt 2-6) did not resolve this. However, less stringent instructions (prompts 7-8) led to correct answers for intermittent clinical signs, although issues remained with historic and resolved clinical signs. Given the priority of

accurately capturing intermittent clinical signs, the instructions below, was selected for subsequent experiments:

- Current or recent signs: If the text indicates that the clinical sign is currently or recently present, answer TRUE.
- Recent occurrence: If the text indicates that the clinical sign occurred recently, answer TRUE, even if the record specifically states that the sign is not currently present.
- Historic signs: If the text indicates that a clinical sign was present at a historic date (not recent) and the clinical problem is either not mentioned in the current status, or mentioned as absent in the current status, answer FALSE.
- Uncertainty: If you are 50/50 on whether to answer TRUE or FALSE, answer TRUE.
- Provide an answer: Always provide an answer. Answers can be only TRUE or FALSE.

Supplementary Material S5: Methods for assessing compliance with instructions

Compliance with output format instructions was evaluated using automated checks built into the API call scripts. These checks verified whether an output was provided for each question and EHR, whether the output was correctly formatted in JSON and whether it contained all the specified fields. To assess compliance with the instructions to always provide a true or false answer for classification questions, a custom R script was used to identify any forbidden NA values. For citations, compliance was evaluated using a custom R script that verified whether the citations adhered to the specified format and compared them with the EHR text for exact matches. For citations that did not exactly match the EHR text, the assessment considered the nature of the discrepancy, whether it altered the meaning of the citation, and if so, whether this change affected the associated classification response.
