# Supplementary material for: Classification performance and reproducibility of GPT-4 omni for information extraction from veterinary electronic health records
Source: Front Vet Sci. 2025 Jan 16;11:1490030. doi: 10.3389/fvets.2024.1490030 (PMC11780673; doi:10.3389/fvets.2024.1490030)
Supplement: Supplementary Presentation 2 — All figures compiled to a PDF. The order of Fig S1 and S2 changed (to comply wth changes in the text) Lgeneds updated to comply with format of main figures. [file Presentation_2.pdf]

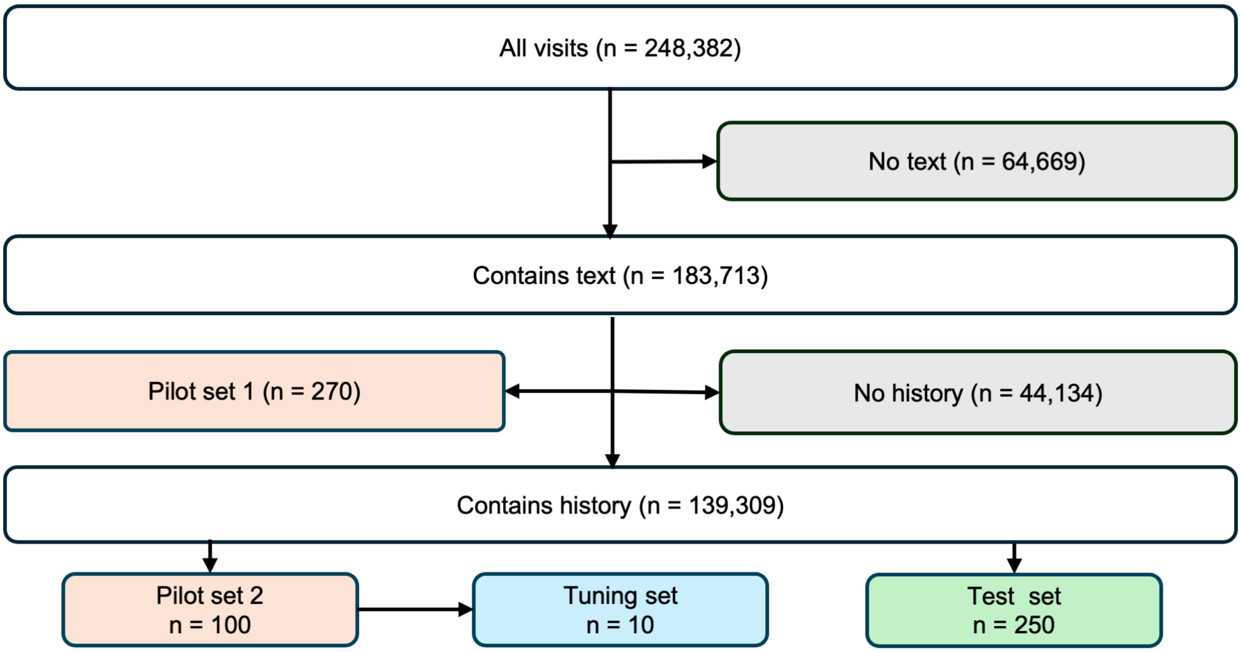

SUPPLEMENTARY FIGURE S1

Flowchart for electronic health records (EHRs). Out of all electronic health records from feline visits between 1985 and 2023, EHRs for the test set were sampled from all EHRs containing history that had not been used in any pilot or tuning sets. White, sample pools; Grey, exclusions; Peach, pilot sets; Blue, tuning set; Green, test set.

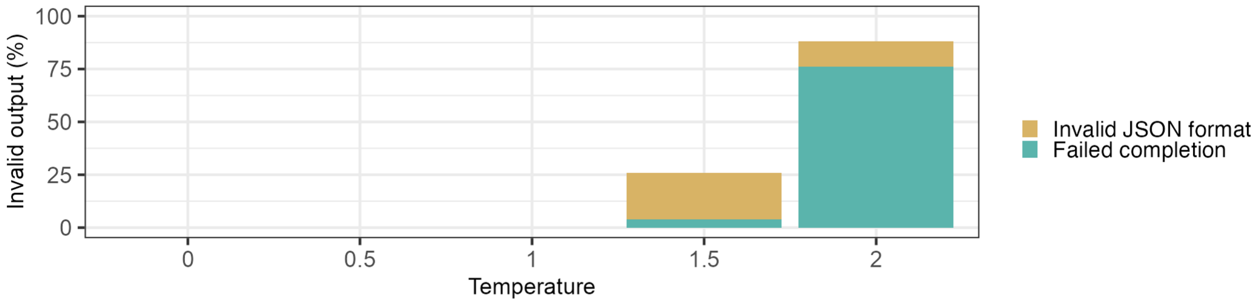

SUPPLEMENTARY FIGURE S2

Invalid output formats in tuning set. A preliminary experiment examined frequencies of invalid output formats for GPT-4 omni (GPT-4o) at temperatures between 0 and 2. High frequencies of Invalid JSON format and complete output failures (due to invalid Unicode) by GPT-4o informed the decision to restrict the temperature settings examined in the test set to values between 0 and 1.

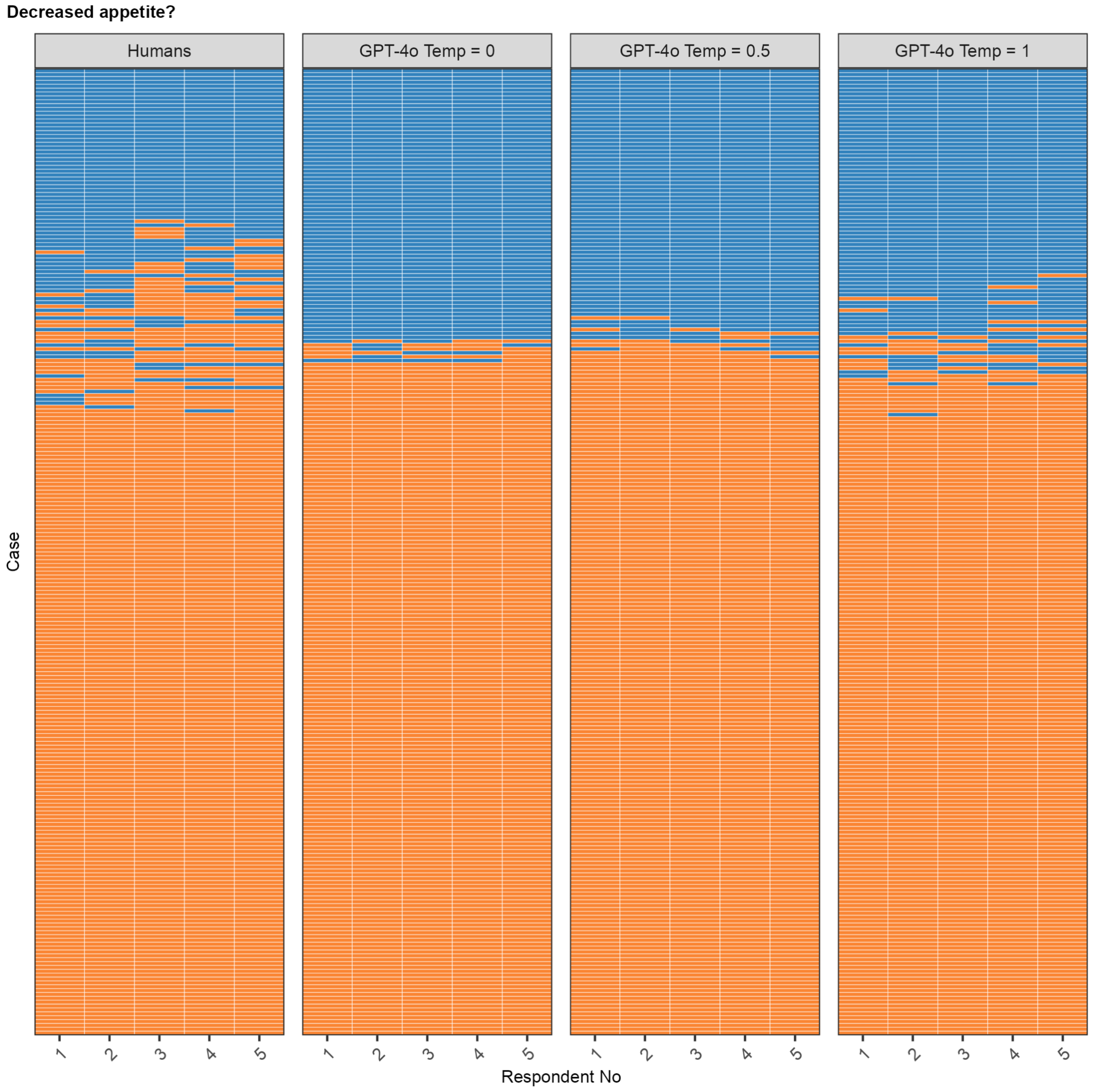

SUPPLEMENTARY FIGURE S3

Decreased appetite? - responses for the test set. Five humans and five repeated runs of GPT-4 omni per temperature setting were tasked with identifying whether each record mentioned the presence of “Decreased appetite”. GPT-4o, GPT-4 omni; Temp, temperature, Blue, true; Orange, false.

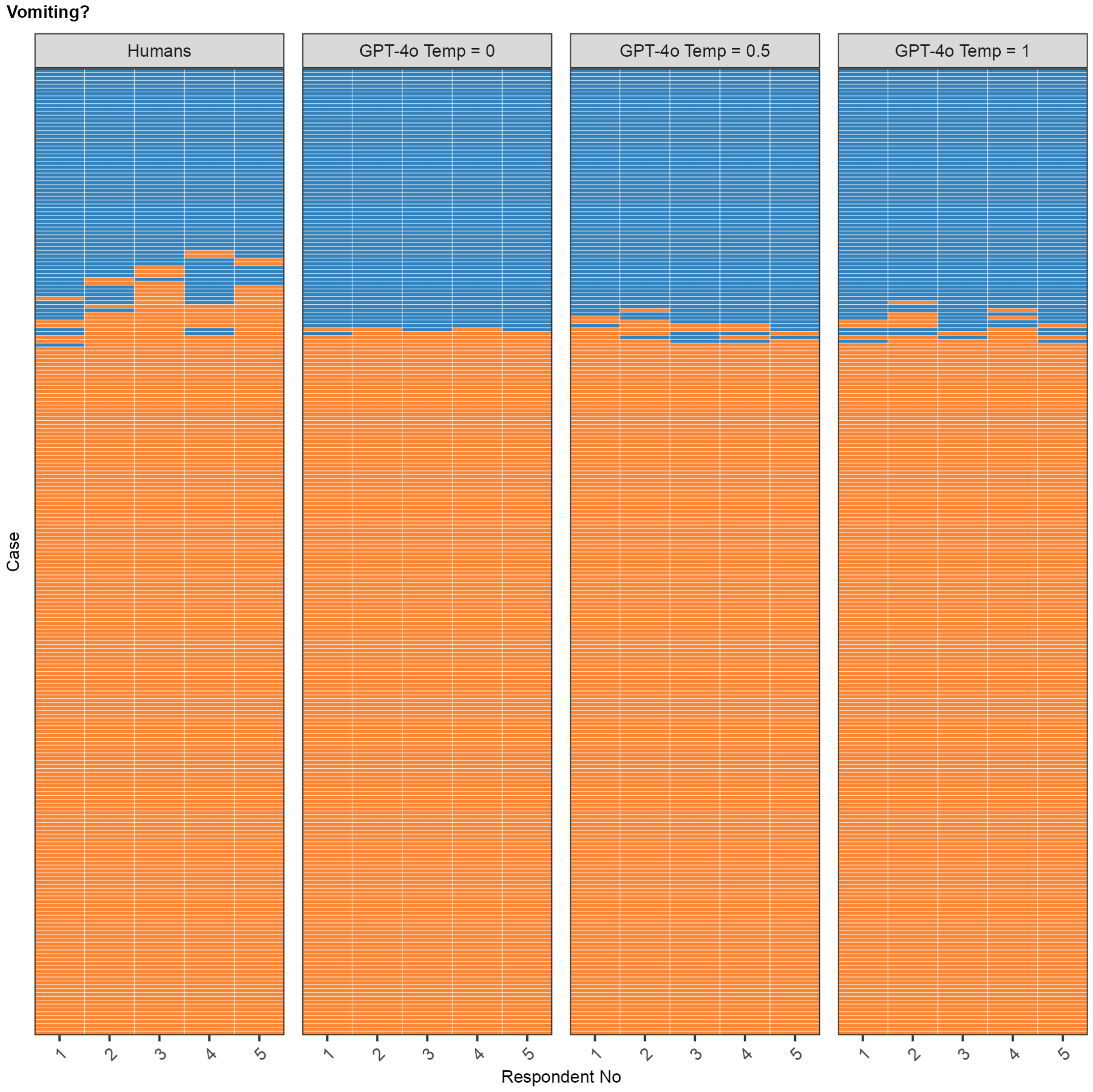

SUPPLEMENTARY FIGURE S4

Vomiting? - responses for the test set. Five humans and five repeated runs of GPT-4 omni per temperature setting were tasked with identifying whether each record mentioned the presence of “Vomiting”. GPT-4o, GPT-4 omni; Temp, temperature; Blue, true; Orange, false

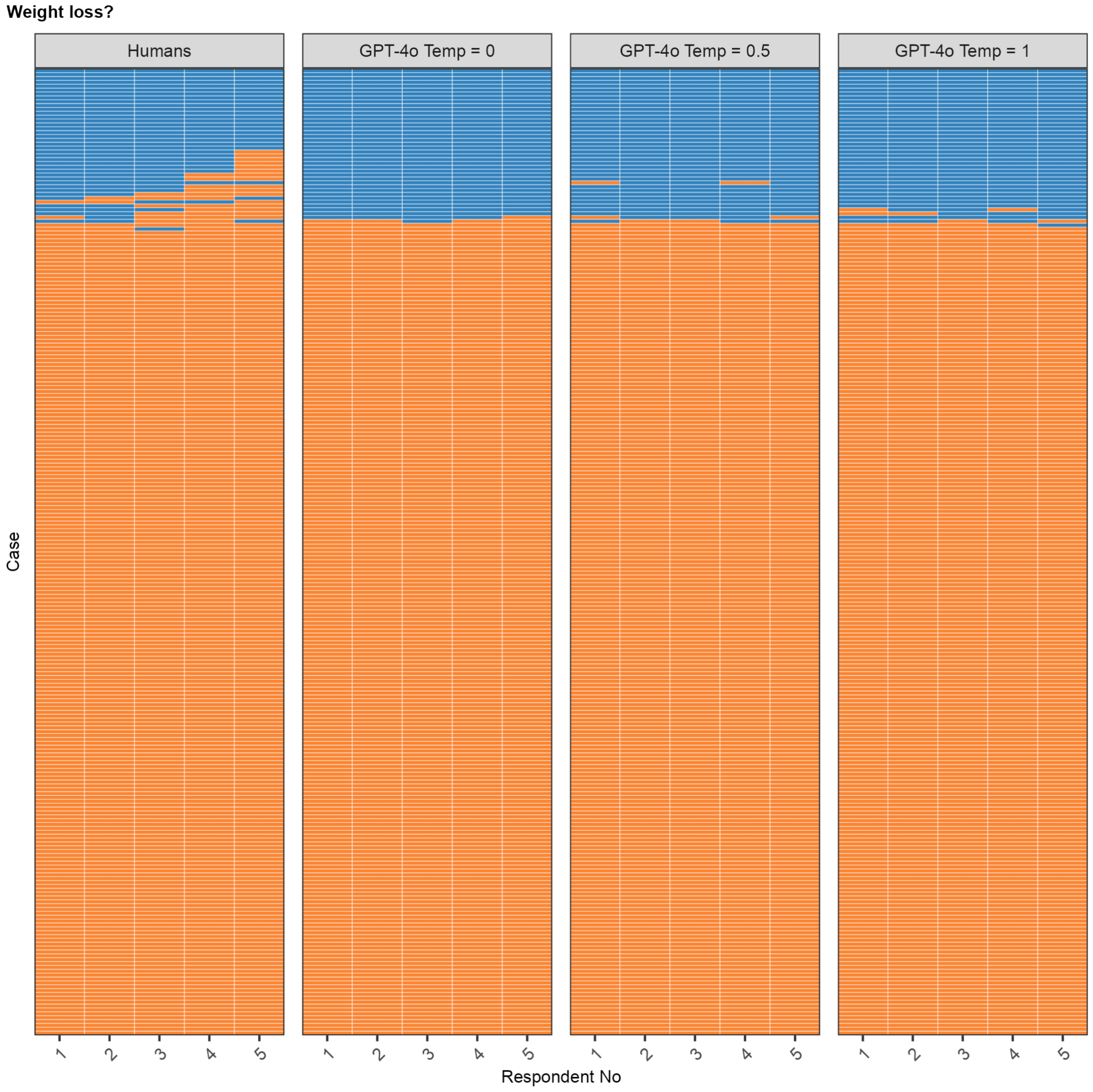

SUPPLEMENTARY FIGURE S5

Weight loss? - responses for the test set. Five humans and five repeated runs of GPT-4 omni per temperature setting were tasked with identifying whether each record mentioned the presence of “Weight loss”. GPT-4o, GPT-4 omni; Temp, temperature; Blue, true; Orange, false.

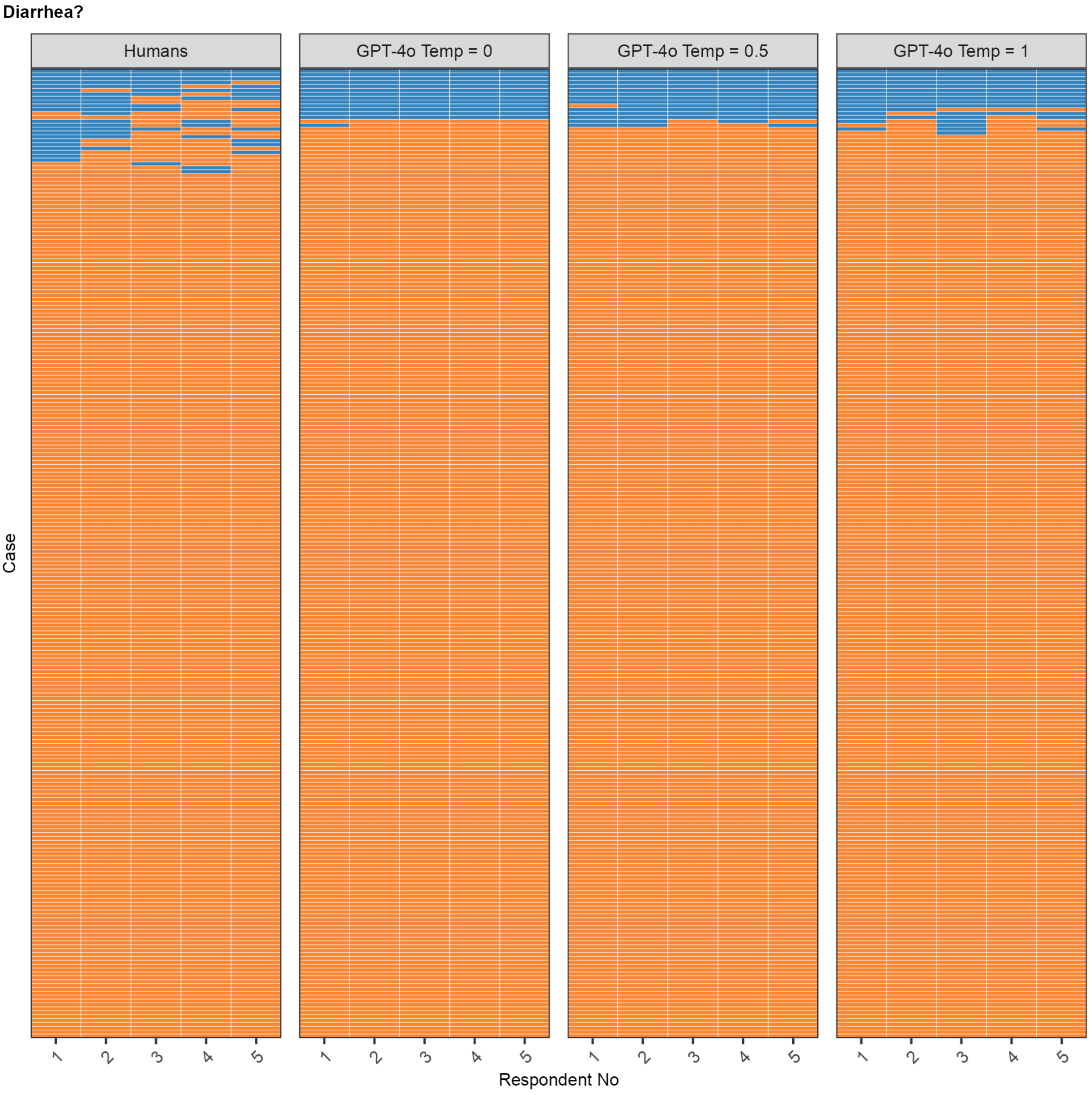

SUPPLEMENTARY FIGURE S6

Diarrhea? - responses for the test set. Five humans and five repeated runs of GPT-4 omni per temperature setting were tasked with identifying whether each record mentioned the presence of “Diarrhea”. GPT-4o, GPT-4 omni; Temp, temperature; Blue, true; Orange, false

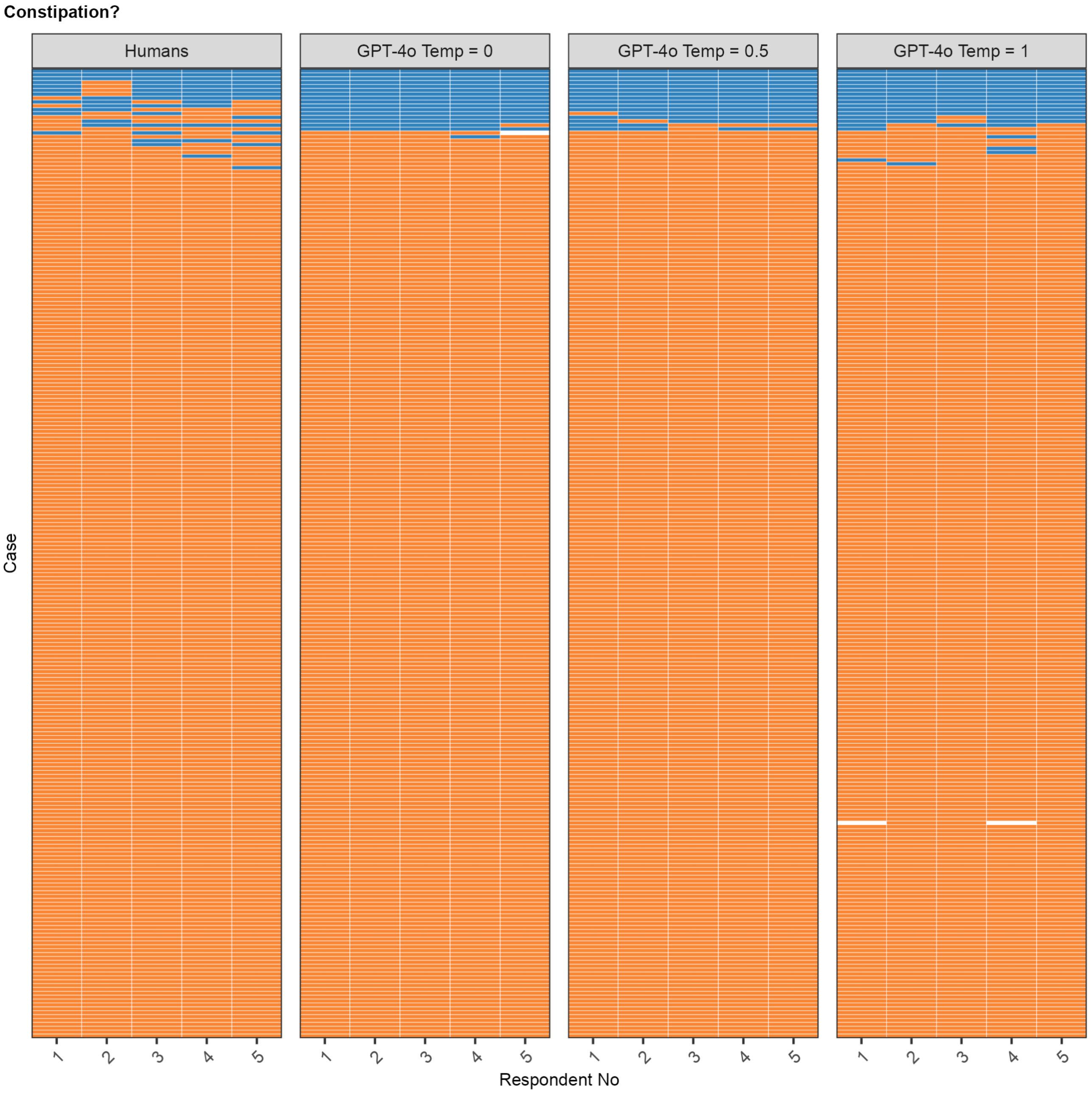

SUPPLEMENTARY FIGURE S7

Constipation? - responses for the test set. Five humans and five repeated runs of GPT-4 omni per temperature setting were tasked with identifying whether each record mentioned the presence of “Constipation”. GPT-4o, GPT-4 omni; Temp, temperature; Blue, true; Orange, false; White, NA

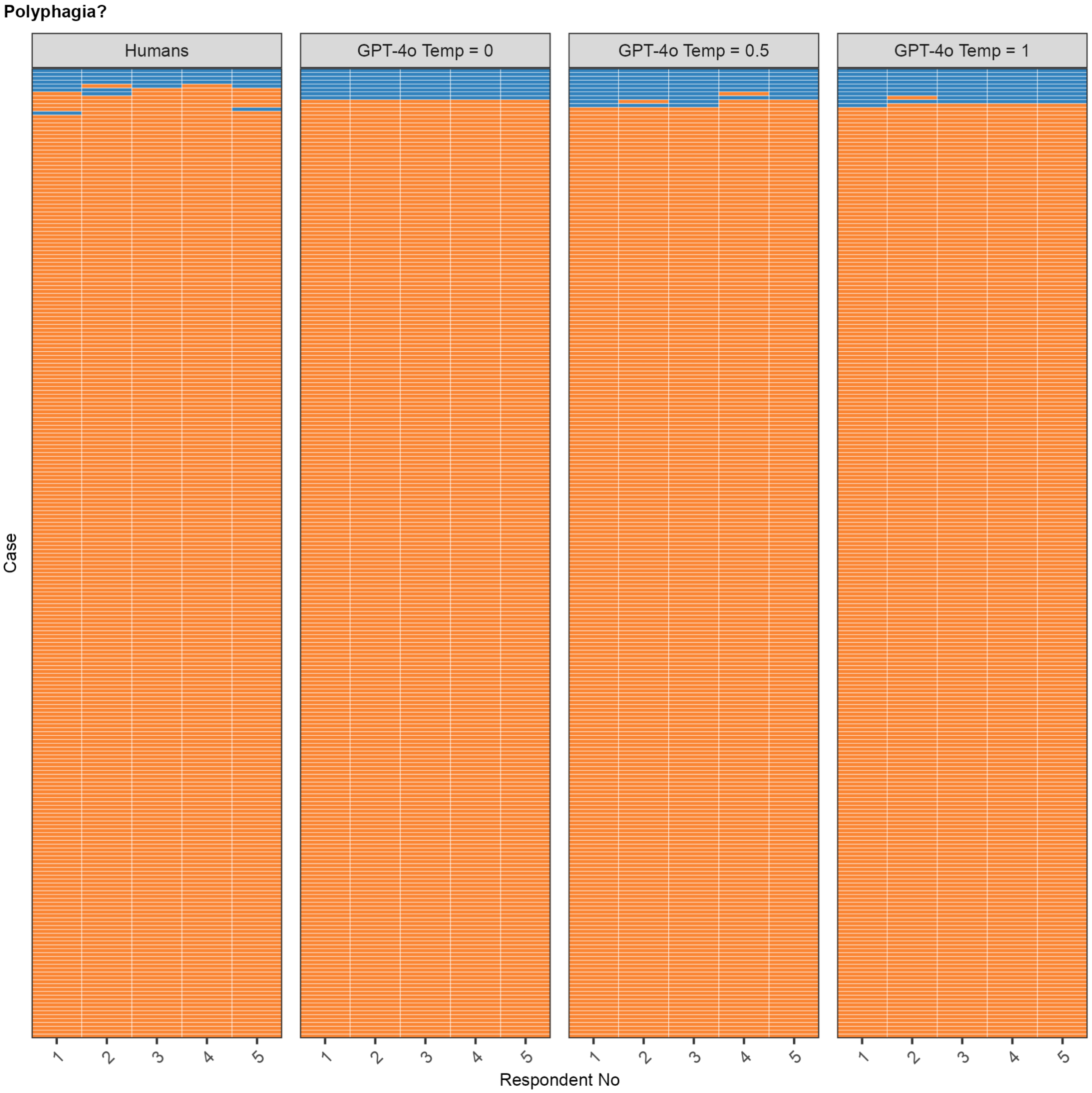

SUPPLEMENTARY FIGURE S8

Polyphagia? - responses for the test set. Five humans and five repeated runs of GPT-4 omni per temperature setting were tasked with identifying whether each record mentioned the presence of “Polyphagia”. GPT-4o, GPT-4 omni; Temp, temperature; Blue, true; Orange, false
