# Supplementary material for: Classification performance and reproducibility of GPT-4 omni for information extraction from veterinary electronic health records
Source: Front Vet Sci. 2025 Jan 16;11:1490030. doi: 10.3389/fvets.2024.1490030 (PMC11780673; doi:10.3389/fvets.2024.1490030)
Supplement: Supplementary Presentation 3 — Saved as PDF & changes in legends to coply with format. for main paper. [file Presentation_3.pdf]

SUPPLEMENTARY TABLE S1

Estimated prevalence of six clinical signs in feline electronic health records UC Davis 1985-2023. Prevalence estimates were calculated for mentions of clinical signs in feline electronic health records (EHRs) using different methods, subset of EHRs, and fields of EHRs.

**Method**	**Set**	**Set size**	**Field**	**Decreased appetite**	**Vomiting**	**Weight loss**	**Diarrhea**	**Constipation**	**Polyphagia**
Keyword search	Full database[3]	183,713	All	6.5%	26.3%	11.2%	16.6%	2.5%	0.9%
Human review[1]	Pilot free text	34	All	23.5%	35.3%	17.6%	20.6%	5.9%	2.9%
Keyword search	Full database[4]	139,579	History	4.1%	30.1%	8.4%	17.3%	2.1%	0.6%
Human review[1]	Pilot history	100	History	16.0%	23.0%	6.0%	14.0%	3.0%	4.0%
Human review[2]	Test set	250	History	24.8%	24.0%	13.6%	5.6%	4.8%	2.0%

[1]One human (JMW) [2]Five humans (majority opinion) [3]Excluding EHRs lacking free text [4]Excluding EHRs lacking history

SUPPLEMENTARY TABLE S2

Average large language model (LLM) performance compared to human majority opinion, across six clinical signs by model and temperature.

**Model**	**Temp**	**Sensitivity Median (IQR)**	**Specificity Median (IQR)**	**PPV Median (IQR)**	**NPV Median (IQR)**	**F1 score Median (IQR)**	**Balanced accuracy Median (IQR)**
GPT-4o	0	97.0 (93.0-99.3)	98 (96-99)	81 (71-85)	100 (99-100)	85 (78-90)	96 (95-98)
GPT-4o	0.5	99 (89-100)	98 (96-98)	82 (70-85)	100 (99-100)	87 (76-92)	97 (93-99)
GPT-4o	1	99 (94-100)	98 (96-98)	80 (74-84)	100 (99-100)	86 (76-92)	97 (95-99)
GPT-3.5 Turbo	0	82 (79-85)	99 (98-99)	84 (78-92)	99 (97-99)	81 (79-86)	91 (89-92)
GPT-3.5 Turbo	0.5	82 (79-85)	99 (98-99)	82 (78-91)	99 (97-99)	82 (79-85)	91 (89-91)
GPT-3.5 Turbo	1	84 (81-86)	99 (98-99)	85 (76-94)	99 (97-99)	81 (80-87)	92 (90-92)

GPT-4o, GPT-4 omni; IQR, interquartile range; PPV, positive predictive value; NPV, negative predictive value; Temp, temperature

SUPPLEMENTARY TABLE S3

Cohen's Kappa for pairs of human respondents and repeated runs of GPT-4 omni at different temperatures.

**Respondent**	**Temp**	**Median (IQR)**	**1 vs 2**	**1 vs 3**	**1 vs 4**	**1 vs 5**	**2 vs 3**	**2 vs 4**	**2 vs 5**	**3 vs 4**	**3 vs 5**	**4 vs 5**
GPT-4o	0	0.98 (0.98-0.99)	0.98	0.99	0.99	0.98	0.98	0.99	0.98	0.99	0.98	0.98
GPT-4o	0.5	0.96 (0.95-0.96)	0.96	0.95	0.95	0.95	0.96	0.95	0.96	0.97	0.96	0.96
GPT-4o	1	0.93 (0.92-0.93)	0.93	0.94	0.91	0.94	0.92	0.92	0.93	0.93	0.95	0.93
Humans	NA	0.8 (0.78-0.81)	0.88	0.78	0.80	0.78	0.79	0.81	0.75	0.81	0.83	0.8

GPT-4o, GPT-4 omni; IQR, interquartile range; Temp, temperature

SUPPLEMENTARY TABLE S4

Compliance with instructions for human respondents and GPT-4o at different temperatures. Frequencies of compliant responses to questions about six clinical signs in 250 electronic health records, for five repeated runs per temperature (or five human respondents

Respondent	Temp	Compliance with output format (%)	Compliance with classification instructions (%)	Compliance with citation instructions (%)
GPT-4o	0	99.9 (7,494/7,500)	99.99 (7,499/7,500)	97.4 (7,302/7,500)
GPT-4o	0.5	100 (7,500/7,500)	100 (7,500/7,500)	95.2 (7,142/7,500)
GPT-4o	1	99.4 (7,452/7,500)	99.97 (7,498/7,500)	90.5 (6,785/7,500)
Humans	NA	NA	NA	96.7 (7,255/7,500)

GPT-4o, GPT-4 omni; Temp, temperature.

SUPPLEMENTARY TABLE S5

Discrepancies between citation and electronic health record texts. Frequencies of different types of discrepancies between citations and electronic health records, to questions about six clinical signs in 250 electronic health records, for five repeated runs per temperature (or five human respondents).

Respondent	Quotation, capitalization, punctuation or spacing (%)	Shortening of the text (%)	Paraphrasing (%)	Inclusion of question or field name in the response (%)
GPT-4o at temperature 0	1.5 (113/7,500)	0.7 (56/7,500)	0.3 (19/7,500)	0.1 (10/7,500)
Humans	2.8 (209/7,500)	0.1 (8/7,500)	0	0.4 (27/7,500)

GPT-4o, GPT-4 omni`
